# Female with Vaginal Bleeding

**DOI:** 10.5811/cpcem.2020.8.48627

**Published:** 2020-10-20

**Authors:** Mark Quilon, Alec Glucksman, Gregory Emmanuel, Josh Greenstein, Barry Hahn

**Affiliations:** * Staten Island University Hospital, Department of Emergency Medicine, Staten Island, New York; † Massachusetts General Hospital, Department of Radiology, Boston, Massachusetts

**Keywords:** ultrasound, pregnancy

## Abstract

**Case Presentation:**

A 24-year-old pregnant female presented to the emergency department with lower abdominal cramping and vaginal bleeding. A point-of-care ultrasound demonstrated a calcified yolk sac.

**Discussion:**

When identified, calcification of the yolk sac in the first trimester is a sign of fetal demise. It is important for an emergency physician to be aware of the various signs and findings on point-of-care ultrasound and be familiar with the management of these pathologies.

## CASE PRESENTATION

**Image 1:**
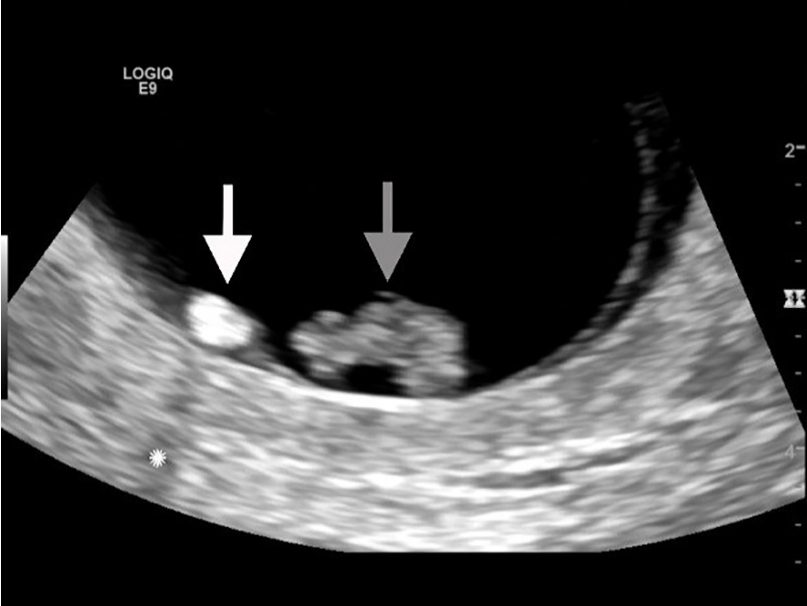
Close-up view of transvaginal sonogram demonstrating a fetal pole (gray arrow) with an adjacent echogenic yolk sac (white arrow), along with posterior acoustic shadowing (asterisk).

**Image 2:**
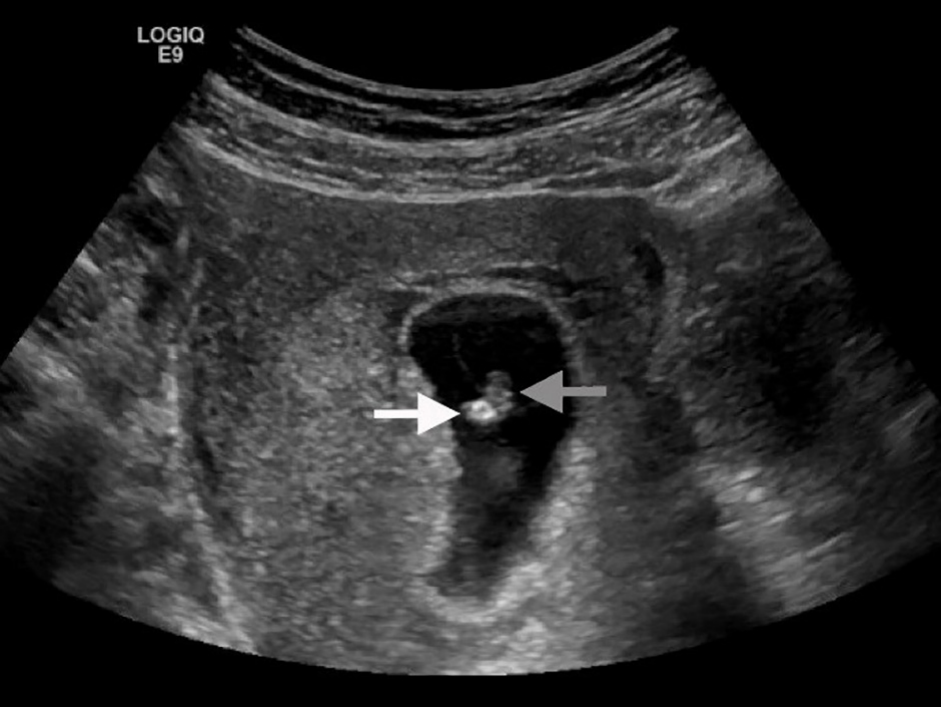
Transvaginal sonogram demonstrating a fetal pole (gray arrow) with an adjacent echogenic yolk sac (white arrow).

A 24-year-old female presented to the emergency department (ED) with three days of lower abdominal cramping and vaginal spotting. Physical examination demonstrated suprapubic tenderness, scant blood in the vaginal vault, and closed cervix. The urine pregnancy test was positive. An ED point-of-care, pelvic ultrasound identified a fetal pole with an adjacent echogenic yolk sac (Images 1 and 2, video). Fetal cardiac activity was not appreciated.

## DISCUSSION

Calcification of the yolk sac is likely due to dystrophic calcification, in which dead tissue undergoing necrosis accumulates calcium.^1^ Calcification of the yolk sac in the first trimester is a sign of fetal demise.^2^ Ultrasound will show increased echogenicity within the gestational sac. The normal ring-like appearance of the yolk sac will instead resemble a hyperechoic, dense, irregular structure, along with posterior acoustic shadowing and possibly comet-tail artifact.^3^,^4^

Calcified yolk sacs are usually indicative of a loss of fetal cardiac activity before 12 weeks of gestation. Greater than 80% of miscarriages occur in the first trimester, and calcification of the yolk sac may be seen at that time. However, the prevalence is currently unknown. Risk factors are the same as those that are commonly associated with miscarriage, including previous miscarriage, fetal chromosomal abnormalities, advanced maternal age, and diabetes. Signs and symptoms include abdominal pain or cramps and vaginal bleeding. Physical findings include abdominal tenderness and blood in the vaginal vault with an open or closed cervix. Treatment is expectant management and symptomatic therapy. Outpatient obstetrical follow-up with ultrasonography at 7–14 days to assess the pregnancy for viability is generally appropriate. A calcified yolk sac does not confer any higher risk of future miscarriage or infertility, similar to other first-trimester spontaneous abortions.^5^

CPC-EM Capsule

What do we already know about this clinical entity?

*A calcified yolk sac is a sonographic sign associated with fetal demise and inevitable abortion*.

What is the major impact of the image(s)?

*This image can help physicians distinguish fetal demise from other signs of early pregnancy*.

How might this improve emergency medicine practice?

*Identification of a calcified yolk sac can help manage patient expectations and allow the emergency medicine clinician to counsel the patient, and arrange appropriate follow up*.

## Supplementary Information

Video Transvaginal sonogram demonstrating a fetal pole (solid arrowhead) with an adjacent echogenic yolk sac (hollow arrow).
